# Topics of the Month

**Published:** 1896-05

**Authors:** 


### TOPICS OF THE MONTH.

In referring to the typhoid fever epidemic at Elmira in the April issue of the Journal, it was far from our purpose to cast any reflection on the health department of that city, so ably presided over by Dr. Wm. C. Wey. Nevertheless, we fear that a careful reading of our paragraph might lead to a possible construction as of censure. As a matter of fact, we are informed that the Elmira health board did take timely action with reference to the water supply of that city. Through its recommendation a consulting engineer was employed and an elaborate report was made showing the sources of impurity and recommending a remedy; but there was delay in action, a circumstance for which the health board was in no wise responsible, and direful results were the consequence. We understand that the evil complained of has been corrected and that in future there will be no contamination of the water supply of Elmira.

---

The effects of tuberculosis on dairy cattle has attracted the attention of sanitarians and public health authorities for some time. A committee of the state board of health has conferred with a similar committee of the board of health of the city of New York and a report has been published for distribution among the farmers. The report states that more than one-seventh of all deaths occurring in human beings throughout the civilised world is caused by tuberculosis, and it is estimated that more than one-fourth of all deaths occurring during adult life is due to it and that nearly one-half of the entire population of the world at some time in life acquires it.

The report shows a distinct relation between tuberculosis in the human being and that of the tuberculous cow, and that the milk from infected cows is an active source of the disease. Among stall-fed dairy cows from 5 to 50 per cent. are sometimes found to be affected with the disease. Tuberculous animals are also frequently killed for food. Their flesh sometimes contains the germs, and if not thoroughly cooked is capable of transmitting the disease. Boiling the milk and thoroughly cooking the meat destroy the germs.

The report further shows the prevalence of the disease in this state and it affirms that tuberculosis is now so widespread that it is difficult to prevent the infection of herds even with the best of care. The committee states that destruction is the only proper mode of disposal of diseased animals, and that their sale for food or their use for milking is not only dishonorable but may become criminal. To suppress the disease the committee suggests the use of tuberculin and the destruction of cattle shown by the test to be diseased. The committee recommends the state to make an immediate appropriation of $300,000 to secure the suppression of the disease.

---

A DANGEROUS bill has been introduced in the assembly, the purpose of which is to incorporate the Optical Society of the State of New York. This society is not composed of physicians, but of opticians, upon whom it confers the exclusive right to issue licenses to persons whom it designates refracting opticians, giving them the right to fit glasses for various imperfections of vision, a service that none but educated physicians is competent to perform. Last year, in spite of the greatest watchfulness, a bill crept through which conferred medical or surgical rights upon chiropodists, but it is hoped that a similar evil will not happen this year through the apathy of the medical profession. So far we have been fortunate in escaping serious damage through the enactment of unwise medical laws, and it is to be ardently hoped that the last hours of the present legislature will not leave to the medical profession a legacy of unhappy surprises. Least of all should this optical bill be allowed to become a law.

---

AT A MEETING of the Philadelphia County Medical Society, held April 15, 1896, Drs. John B. Roberts, James C. Wilson and William M. Welch were appointed a committee to urge the members of the American Medical Association to favor the holding of a semicentennial celebration of its organisation. The society also instructed its delegates to invite the association to hold the meeting of 1897, which will be the semicentennial, in the city of Philadelphia. The traditions of Philadelphia, medically, historically and socially, are such as to render it an appropriate city in which to hold the proposed jubilee. We hope our Philadelphia friends of the committee named may be successful in securing the object for which they are earnestly laboring.

---

DR. ERNEST WENDE, chairman, has issued a call convening the committee appointed by the Medical Society of the County of Erie, at its seventy-fifth anniversary meeting, to consider the feasibility of establishing a permanent home for the several medical bodies in Erie county. The meeting will be held at the office of the health commissioner, municipal building, Buffalo, Friday, May 1, 1896, at 3 o'clock P. M. An attendance of every member of the committee is urged, as ways and means will be considered, an address formulated and other important subjects connected with the scheme discussed. The committee is constituted as follows: Drs. Ernest Wende, chairman; J. G. Thompson, Angola; Herman Mynter, Buffalo; Henry Lapp, Clarence, and Walter D. Greene, Buffalo.

